# Comorbidity of self-harm and disordered eating in young people: Evidence from a UK population-based cohort

**DOI:** 10.1016/j.jad.2020.12.053

**Published:** 2021-03-01

**Authors:** Naomi Warne, Jon Heron, Becky Mars, Paul Moran, Anne Stewart, Marcus Munafò, Lucy Biddle, Andy Skinner, David Gunnell, Helen Bould

**Affiliations:** a Centre for Academic Mental Health, Population Health Sciences, Bristol Medical School, University of Bristol, Bristol, UK; b Centre for Public Health, Population Health Sciences, Bristol Medical School, University of Bristol, Bristol, UK; c NIHR Biomedical Research Centre, University Hospitals Bristol NHS Foundation Trust, University of Bristol, Bristol, UK; d NIHR Applied Research Collaboration (ARC) West at University Hospitals Bristol NHS Foundation Trust, Bristol, UK; e Department of Psychiatry, University of Oxford, Oxford, UK; f Oxford Health NHS Foundation Trust, Oxford UK; g School of Psychological Science, University of Bristol, Bristol, UK; h MRC Integrative Epidemiology Unit, University of Bristol Medical School, Bristol, UK; i Gloucestershire Health and Care NHS Foundation Trust, Gloucester, UK

**Keywords:** ALSPAC, Self-harm, Disordered eating, Comorbidity, Epidemiology

## Abstract

**Background:**

Self-harm and eating disorders are often comorbid in clinical samples but their co-occurrence in the general population is unclear. Given that only a small proportion of individuals who self-harm or have disordered eating present to clinical services, and that both self-harm and eating disorders are associated with substantial morbidity and mortality, it is important to study these behaviours at a population level.

**Methods:**

We assessed the co-occurrence of self-harm and disordered eating behaviours in 3384 females and 2326 males from a UK population-based cohort: the Avon Longitudinal Study of Parents and Children (ALSPAC). Participants reported on their self-harm and disordered eating behaviours (fasting, purging, binge-eating and excessive exercise) in the last year via questionnaire at 16 and 24 years. At each age we assessed how many individuals who self-harm also reported disordered eating, and how many individuals with disordered eating also reported self-harm.

**Results:**

We found high comorbidity of self-harm and disordered eating. Almost two-thirds of 16-year-old females, and two-in-five 24-year old males who self-harmed also reported some form of disordered eating. Young people with disordered eating reported higher levels of self-harm at both ages compared to those without disordered eating.

**Limitations:**

We were not able to measure whether participants identified their disordered eating as a method of self-harm.

**Conclusions:**

Self-harm and disordered eating commonly co-occur in young people in the general population. It is important to screen for both sets of difficulties to provide appropriate treatment.

## Highlights

• Self-harm and disordered eating commonly occurred in 16- and 24-year-olds.
• Females typically had higher rates of comorbidity than males.
• Almost two-thirds of females self-harming reported some form of disordered eating.
• Screening for both self-harm and disordered eating in clinical settings is important.

## 1 Introduction

Eating disorders and self-harm are serious health problems in young people, and are associated with significant functional impairment and mortality (Hawton et al., 2012; Treasure et al., 2020). They are phenotypically distinct - eating disorders involve weight-control behaviours, abnormal eating and over-evaluation of weight and shape (Treasure et al., 2020), whereas self-harm involves intentionally harming oneself, with or without suicidal intent (Hawton et al., 2012). However, they commonly co-occur in clinical populations: 14-68% of patients with eating disorders report self-harm and 54-61% of patients who self-harm also have an eating disorder diagnosis (Svirko and Hawton, 2007). More recently, a meta-analysis found 21.8% of patients with anorexia nervosa and 32.7% of patients with bulimia nervosa had a lifetime history of self-harm without suicidal intent (Cucchi et al., 2016). This comorbidity may reflect common risk factors, such as emotion dysregulation or impulsivity (Svirko and Hawton, 2007). However, few studies have assessed the comorbidity of disordered eating and self-harm in non-clinical samples. Such research is important as only a small proportion of individuals who self-harm or have disordered eating present to clinical services (Hawton et al., 2012; Treasure et al., 2020), so comorbidity in clinical samples may not reflect true levels of comorbidity in the general population.

Studies using non-clinical samples to assess the co-occurrence of disordered eating and self-harm have predominantly focused on either university students or adolescents. For instance, 50.8% of female university students who self-harmed reported a possible eating disorder, and 20.1% with a possible eating disorder reported self-harm (Wright et al., 2009). Furthermore, university students self-harming more than once are more likely to have disordered eating (45.2%) than students who only self-harm once (24.8%) and students who have not self-harmed (19.6%) (Whitlock et al., 2006). Studies using adolescent participants, recruited and tested in school settings, have found that there are higher levels of disordered eating in adolescents who report self-harm compared to those who do not (Brausch and Boone, 2015; Ross et al., 2009). In a Swedish community sample of adolescents, eating disorder risk behaviours are positively correlated with levels of self-harm (Bjärehed and Lundh, 2008), and adolescent girls who purge reported greater levels of self-harm cross-sectionally and longitudinally in comparison to adolescent girls without disordered eating and with other clusters of disordered eating (Viborg et al., 2018). Although these studies are informative, unselected samples are required to understand how self-harm and disordered eating co-occur in the general population over the adolescent to young adult age range, during which rates of both may vary (Hawton et al., 2012; Moran et al., 2012; Treasure et al., 2020; Ward et al., 2019).

In this study, we examine the co-occurrence of self-harm and disordered eating at both 16 and 24 years in a UK population-based cohort.

## 2 Methods

Data were from a prospective birth cohort: The Avon Longitudinal Study of Parents and Children (ALSPAC) (Boyd et al., 2013; Fraser et al., 2013; Northstone et al., 2019). ALSPAC recruited pregnant women with expected delivery dates between April 1991 and December 1992 in Avon, UK (core sample n=13,988 alive at 1 year). Ethics approval for the study was obtained from the ALSPAC Ethics and Law Committee and the Local Research Ethics Committees*.* The study website contains details of ALSPAC data: http://www.bristol.ac.uk/alspac/researchers/our-data.

We conducted our analysis on an imputed dataset of 5710 individuals (3384 females, 2326 males) who completed self-harm and disordered eating questionnaires at age 16 and/or 24 years (see Supplementary Material for full details on multiple imputation). *Disordered eating behaviours* in the last year were assessed on adapted Youth Risk Behaviour Surveillance System questions (Kann et al., 1996). Behaviours included *fasting* (not eating for at least one day), *purging* (vomiting or taking laxatives/other medicines), and *excessive exercise* (that frequently interfered with daily routine/work) in order to lose weight or avoid gaining weight, as well as *binge-eating* (eating a very large amount of food, with loss of control, in a short period of time). Behaviours were considered present if endorsed at any frequency in the last year. Our primary variable of interest was *any disordered eating* (any of the aforementioned behaviours); we also present data for individual behaviours and *any disordered eating at DSM-5 frequency* (any of the behaviours at least once a week). For *self-harm* in the last year, participants were asked adapted Child and Adolescent Self-Harm in Europe study questions (Madge et al., 2008) on whether they had hurt themselves on purpose in any way (regardless of suicidal intent), and when this occurred. Questions and variable coding are available in Table S1.

Analyses were a priori stratified by gender. At each age, we assessed 1) the proportion of individuals reporting disordered eating and self-harm in the sample; 2) what proportion of individuals with disordered eating also reported self-harm (compared to those without disordered eating); and 3) what proportion of individuals who self-harmed also reported disordered eating (compared to those not self-harming). Results were consistent between imputed and complete case analysis (Tables S2, S4 and S5). We also explored a possible dose-response effect of self-harm in complete cases by comparing rates of any disordered eating in individuals with no self-harm, a single episode of self-harm and repeated instances (≥2) of self-harm in line with Whitlock et al. (2006).

## 3 Results

Prevalence of individual disordered eating and self-harm behaviours varied by gender and by age (Table 1 and Table S2). At age 16, 32.7% of females and 7.6% of males reported some form of disordered eating in the past year, and 15.3% of females and 5.4% of males reported self-harm in the past year. The most common form of disordered eating at age 16 was fasting (20.7%) for females, and binge-eating (4.3%) for males. Compared to age 16, age 24 prevalence of any past-year disordered eating increased for both females (36.9%) and males (19.2%), but self-harm decreased in females (9.8%) and males (5.1%) across the same time frame. At age 24, binge-eating was the most common form of disordered eating in both females (24.8%) and males (13.7%).

**Table 1:** Prevalence of disordered eating and self-harm at 16 and 24 years.

	Age 16		Age 24	
	Female (n=3384)	Male (n=2326)	Female (n=3384)	Male (n=2326)
	*% (se)*	*% (se)*	*% (se)*	*% (se)*
Any fasting	20.7% (0.77)	3.4% (0.45)	13.6% (0.67)	6.2% (0.70)
Any purging	9.5% (0.56)	1.9% (0.38)	12.7% (0.67)	3.2% (0.55)
Any binge-eating	16.2% (0.70)	4.3% (0.48)	24.8% (0.86)	13.7% (0.97)
Any excessive exercise	2.1% (0.28)	0.8% (0.27)	1.6% (0.28)	2.0% (0.47)
Any disordered eating behaviour	32.7% (0.89)	7.6% (0.63)	36.9% (0.96)	19.2% (1.02)
Any disordered eating behaviour at DSM 5 level frequency	11.1% (0.61)	2.8% (0.41)	11.3% (0.63)	5.9% (0.66)
Any self-harm	15.3% (0.68)	5.4% (0.53)	9.8% (0.61)	5.1% (0.68)

Note: Proportions are presented for imputed results. For comparison with complete case proportions, see Table S2. se = standard error

**Fig. 1:**
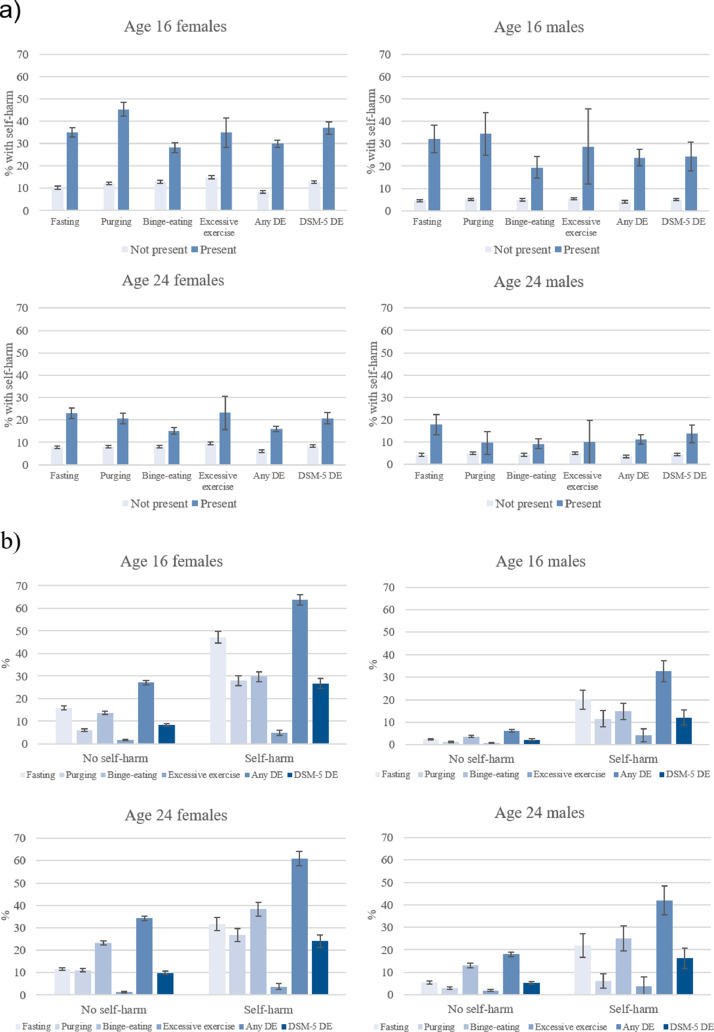
Co-occurrence of self-harm and disordered eating behaviours. Panel (a) Self-harm in individuals with or without disordered eating behaviours. Panel (b) Disordered eating behaviours in individuals with or without self-harm. *Note:* Any DE = any disordered eating (fasting, purging, binge-eating, or excessive exercise); DSM-5 DE = disordered eating (fasting, purging, binge-eating, or excessive exercise) at least once a week (DSM-5 frequency). Error bars indicate standard error.

Co-occurrence of self-harm and disordered eating was common (Fig. 1). In those reporting any disordered eating at age 16, 29.9% of females and 23.7% of males also reported self-harm in the last year, compared to 8.3% of females and 4.0% of males without disordered eating. At age 24, 16.1% of females and 11.1% of males with any disordered eating had also self-harmed, compared to 6.0% of females and 3.6% of males without disordered eating. The eating behaviours associated with the highest levels of self-harm in females were purging at age 16 (45.4%) and excessive exercise at age 24 (23.2%); in males it was purging at age 16 (34.4%) and fasting at age 24 (17.9%).

At age 16, 63.7% of females and 32.7% of males who self-harmed also reported disordered eating, compared to 27.1% of females and 6.1% of males who did not report self-harm. At 24, 60.9% of females who had self-harmed (versus 34.3% who had not) and 41.9% of males who had self-harmed (versus 18.0% who had not) reported disordered eating. In those with self-harm, the highest proportions of concurrent disordered eating behaviours were fasting (47.2%) amongst 16-year-old females, binge-eating (38.4%) in 24-year-old females, fasting (20.1%) in 16-year-old males, and binge-eating (25.1%) amongst 24-year-old males.

There was evidence of a possible dose-response effect in females at age 16 and 24 years, with increasing prevalence of disordered eating in individuals reporting repeated, compared to one-off self-harm; there was limited evidence for this in males (see Supplementary Material Tables S6.1 and S6.2).

## 4 Discussion

In a UK population-based cohort, we found that self-harm and disordered eating behaviours were common, and commonly co-occurred in young people. The prevalence of disordered eating was notably high among females who reported self-harm, affecting nearly two-thirds of young women across both late adolescence and early adulthood. Furthermore, two-in-five 24-year-old males who self-harmed also reported disordered eating. To our knowledge, this is the first study to assess comorbidity of self-harm and disordered eating in both adolescence and young adulthood in the general population.

This high rate of co-occurrence is consistent with findings from selected clinical (Svirko and Hawton, 2007), university student (Whitlock et al., 2006; Wright et al., 2009), and adolescent (Brausch and Boone, 2015; Ross et al., 2009; Viborg et al., 2018) samples. Our study extends these findings to young adults in the community, and demonstrates that they persist across late adolescence into early adulthood. General population data is particularly important since the majority of young people with self-harm or disordered eating do not seek help (Hawton et al., 2012; Treasure et al., 2020). This study provides a more accurate representation of comorbidity in the general population than can be gained from clinical studies. These high levels of comorbidity have important implications for clinical and public health approaches.

Consistent with the previous literature (Brausch and Boone, 2015; Svirko and Hawton, 2007; Wright et al., 2009), we found higher rates of disordered eating among those reporting self-harm than vice versa. We also found higher rates of disordered eating in females with repeated instances of self-harm compared to females with a single instance of self-harm, suggesting a dose-response effect in line with previous research (Whitlock et al., 2006). However, this effect was not seen in males and should be interpreted with caution given the small proportion of individuals with repeated instances of self-harm.

The comorbidity between self-harm and disordered eating may be due to shared risk factors that contribute to the development of both disordered eating and self-harm. A number of potential risk factors (such as impulsivity, emotion dysregulation and dissociation (Svirko and Hawton, 2007)) have been suggested, based on evidence from clinical samples. However, given the small proportions of those with self-harm and disordered eating who present to clinics (Hawton et al., 2012; Treasure et al., 2020), longitudinal studies in population-based samples are needed to assess factors that precede self-harm and disordered eating behaviours. Such research could facilitate early identification of those at high risk of developing self-harm and/or disordered eating and identify modifiable targets for prevention and intervention measures.

The strengths of the current study include the large population-based sample and examination of multiple types of disordered eating over this important life period. There are limitations: firstly, the small number of males reporting behaviours and lack of data at other ages means results may not generalise. Secondly, questions identifying disordered eating may have excluded those with milder, although significant, symptoms. Thirdly, some individuals may self-define their disordered eating as self-harm; we were not able to differentiate when this was the case. Fourthly, the vast majority of participants in ALSPAC were white (>95%; Boyd et al., 2013) so we did not have sufficient numbers of people with different ethnic backgrounds to permit comparisons. Finally, we used an imputed dataset under the assumption data are missing at random, which, if not true, could mean results are biased.

In summary, we found substantial comorbidity between self-harm and disordered eating in adolescents and young adults in the general population. Health professionals should be aware of this comorbidity and ensure that young people presenting with either self-harm or disordered eating are asked about both behaviours in order to provide appropriate treatment and management.

## 5 Data statement

ALSPAC data access is through a system of managed open access. The steps below highlight how to apply for access to the data included in this paper and all other ALSPAC data.

1 Please read the ALSPAC access policy (PDF, 843kB) which describes the process of accessing the data and samples in detail, and outlines the costs associated with doing so.
2 You may also find it useful to browse our fully searchable research proposals database, which lists all research projects that have been approved since April 2011.
3 Please submit your research proposal for consideration by the ALSPAC Executive Committee. You will receive a response within 10 working days to advise you whether your proposal has been approved.

If you have any questions about accessing data or samples, please email alspac-data@bristol.ac.uk (data) or bbl-info@bristol.ac.uk (samples).

## Declaration of Competing Interest

None.

## Appendix Supplementary materials

Image, application 1
