# Supplementary material for: Comorbidity of self-harm and disordered eating in young people: Evidence from a UK population-based cohort
Source: J Affect Disord. 2021 Mar 1;282:386–90. doi: 10.1016/j.jad.2020.12.053 (PMC8150329; doi:10.1016/j.jad.2020.12.053)
Supplement: Supplementary file 1 [file mmc1.docx]

**Comorbidity of self-harm and disordered eating in young people: Evidence from a UK population-based cohort**

**Contents of Supplementary Material**

**Table S1.**

Derivation of self-harm (SH) and disordered eating (DE) variables used in analysis

**Table S2.**

Analyses were repeated across three separate subsamples of the ALSPAC cohort. Sample 1 (n = 1798) consists of individuals who completed self-harm and disordered eating questions at **both** 16 and 24 years. Sample 2 analyses are also based on observed data but in this instance all available data is used – for example analysing all available age 16 responses irrespective of whether the 24-year questionnaire was completed, and using all available data at age 24, even if the age 16 questionnaire was not completed. Sample 2 consists of 4581 individuals at age 16 and 3842 at age 24. Finally, for sample 3, multiple imputation (MI) was employed to boost the sample to those responding at **either** 16 or 24 years (n = 5710). Imputed results (Sample 3) are reported in the main text.

Data were imputed using the “chained equations” or Full Conditional Specification method, using the -ice- package in Stata which makes the **missing at random (MAR)** assumption, namely that any systematic differences between respondents and non-respondents can be adequately explained by other observed data. In addition to the variables required for the substantive analyses, auxiliary variables related to the missing data mechanism and the incomplete model-variables were incorporated. These included self-report data on fasting/purging and bingeing collected at ages 14 and 18 years, BMI collected at age 15 (categorised into <19/19-25/25+), negative mood assessed by the short-form Mood and Feelings Questionnaire at 14 and 18 years, and early experimentation with alcohol and tobacco at age 13. A selection of maternal-report socio-demographic measures such as housing tenure, maternal highest education, social class and family income were also used. 100 imputed datasets were created (a decision informed by studying the Monte Carlo errors for the estimated parameters) and 20 cycles of switching were used. To facilitate gender-specific estimates to be derived, a stratified approach to the imputation was employed, using the by() option. Finally, to mitigate against the danger of perfect prediction given the large number of sparse categorical data analysed, six separate imputation models were derived – one for each of the disordered eating measures. The self-harm data were included in all six imputations.

Table S2 indicates a slight drift upwards in the prevalence of both self-harm and disordered eating at both ages as partial respondents are included, consistent with our expectation that affected individuals are slightly less likely to return questionnaires.

**Tables S3.1 and S3.2**

Tables S3.1 and S3.2 are an alternative way of presenting the MI figures from Table S2. Since observational data are unlikely to be Missing Completely at Random (MCAR) we would anticipate a different mean prevalence for self-harm and disordered eating among non-responders. These two tables show the predicted prevalence of self-harm and disordered eating to be moderately higher among non-responders – assuming the imputation model to be correct and the Missing at Random (MAR) assumption to be justified. Whilst the Risk Ratio estimates suggest a stronger relationship between SH/DE and response in males, these data should be interpreted with caution since the observed prevalence of some disordered eating behaviours was very low.

**Tables S4.1 and S4.2**

Tables S4.1 and S4.2 contain the estimated rates of self-harm across strata of disordered eating for the three analytical subsamples. Since we anticipated prevalence estimates to change due to the missing data mechanism, these tables also contain Risk Ratios in an attempt to capture the SH-DE relationship in a single parameter. Estimates show our conclusions to be robust to the choice of sample used.

Asterisks (“**”) indicate that some estimates of prevalence or Risk Ratios were reliant on cells containing five individuals or fewer. It is an ALSPAC stipulation that such data are not tabulated due to difficulties in maintaining participant anonymity for such rare characteristics.

**Table S5**

Table S5 contain the estimated rates of disordered eating behaviours across strata of self-harm for the three analytical subsamples. Not surprisingly given that the same data has been analysed from two different perspectives, these figures also show a degree of robustness across the samples analysed.

**Tables S6.1 and S6.2**

Tables S6.1 and S6.2 contain descriptive analyses in complete cases examining a dose-response effect of self-harm; namely, rates of any disordered eating in individuals with no self-harm in the last year, a single instance of self-harm and repeated instances (≥2) of self-harm.

**Table S1. Disordered eating and self-harm variables at age 16 and 24 years in Avon Longitudinal Study of Parents and Children (ALSPAC)**

| **Questions** | **Answers** | **Coding** | **Final variable** |
| --- | --- | --- | --- |
| **Age 16** | | | |
| **Fasting (age 16)** | | | |
| During the **past year**, how often did you fast (not eat for at least a day) to lose weight or avoid gaining weight? | 1. Never 2. Less than once a month 3. 1-3 times a month 4. Once a week 5. More than once a week | Any fasting  1 = “no”  2-5 = “yes”  DSM fasting  1-3 = “no”  4-5 = “yes” | **Any fasting (age 16)** |
| **Purging (age 16)** | | | |
| During the **past year**, how often did you make yourself throw up (vomit) to lose weight or avoid gaining weight? | 1. Never 2. Less than once a month 3. 1-3 times a month 4. Once a week 5. 2-6 times a week 6. Every day | Any self-induced vomiting  1 = “no”  2-6 = “yes”  DSM self-induced vomiting  1-3 = “no”  4-6 = “yes” | **Any purging (age 16)**  Any self-induced vomiting  OR  Any laxative/medication use |
| a) During the **past year**, did you take laxatives or other tablets or medicines (diet pills or water tablets) to lose weight or avoid gaining weight?  b) How often? | 1. Yes, laxative 2. Yes, other 3. Never 4. Never 5. Less than once a month 6. 1-3 times a month 7. Once a week 8. 2-6 times a week 9. Every day | Any laxative/medication use  a) 3 = “no”  a) 1-2 = “yes”  OR  b) 2-6 = “yes”  DSM laxative/ medication use  b) 1-3 = “no”  b) 4-6 = “yes”  (“no” if Any laxative/medication use = “no”) |  |
| **Excessive exercise (age 16)** | | | |
| During the **past year**, how often did you do any exercise (going to the gym, brisk walking or any sports activity)? | 1. 5 or more times a week 2. 1-4 times a week 3. 1-3 times a month 4. Less than once a month 5. Never | Any exercise  5 = “no”  1-4 = “yes”  DSM exercise  3-5 = “no”  1-2 = “yes” | **Any excessive exercise (age 16)**  Any exercise to lose weight  AND  Exercise interfered with life  (= 0 if Any exercise = “no”) |
| Did you exercise in order to lose weight or avoid gaining weight? | 1. Yes, sometimes 2. Yes, frequently 3. No | Any exercise to lose weight  3 = “no”  1-2 = “yes” |  |
| Was it difficult for you to do your work or schoolwork because of the amount of time that you were exercising? | 1. Yes, sometimes 2. Yes, frequently 3. No | Exercise interfered with life  1 & 3 = “no”  2 = “yes” |  |
| **Binge eating (age 16)** | | | |
| Sometimes people will go on an ‘eating binge’, where they eat an amount of food that most people would consider to be **very** large, **in a short period of time.** During the **past year**, how often did you go on an eating binge? | 1. Less than once a month 2. 1-3 times a month 3. Once a week 4. More than once a week 5. Never | Any bingeing  5 = “no”  1-4 = “yes”  DSM bingeing  5 & 1-2 = “no”  3-4 = “yes” | **Any binge-eating (age 16)**  Any bingeing  AND  Loss of control |
| Did you feel out of control, like you couldn’t stop eating even if you wanted to? | 1. Yes, usually 2. Yes, sometimes 3. No | Loss of control  3 = “no”  1-2 = “yes” |  |

| **Questions** | **Answers** | | **Coding** | | **Final variable** |
| --- | --- | --- | --- | --- | --- |
| **Any disordered eating (age 16)** | | | | | |
|  |  | |  | | **Any disordered eating (age 16)**  Any fasting OR  Any purging OR  Any binge-eating OR  Any excessive exercise |
| **DSM-5 disordered eating (age 16)** | | | | | |
|  |  | | **DSM purging**  DSM self-induced vomiting OR  DSM laxative/ medication use  **DSM binge-eating**  DSM bingeing AND  Loss of control  **DSM excessive exercise**  DSM exercise AND  Exercise to lose weight AND Exercise interfered with life” | | **DSM-5 disordered eating (age 16)**  DSM fasting  OR  DSM purging  OR  DSM binge-eating  OR  DSM excessive exercise |
| **Self-harm (age 16)** | | | | | |
| When was the **last time** you hurt yourself on purpose? | 1. In the last week 2. More than a week ago but in the last year 3. More than a year ago | | Self-harm past year  3 = “no”  1-2 = “yes” | | **Self-harm (age 16)**  Self-harm past year  (= 0 if self-harm ever = no) |
| Have you ever hurt yourself on purpose in any way (e.g. by taking an overdose of pills, or by cutting yourself)? | 1. Yes 2. No | | Self-harm ever  1 = “yes”  2 = “no” | |  |
| **Age 24** | | | | | |
| **Fasting (age 24)** | | | | | |
| During the **past year**, how often did you fast (not eat for at least a day) to lose weight or avoid gaining weight? | | 1. Never 2. Less than once a month 3. 1-3 times a month 4. Once a week 5. More than once a week | | Any fasting  0 = “no”  1-4 = “yes”  DSM fasting  0-2 = “no”  3-4 = “yes” | **Any fasting (age 24)** |
| **Purging (age 24)** | | | | | |
| During the **past year**, how often did you make yourself throw up to lose weight or avoid gaining weight? | | 1. Never 2. Less than once a month 3. 1-3 times a month 4. Once a week 5. More than once a week | | Any self-induced vomiting  0 = “no”  1-4 = “yes”  DSM self-induced vomiting  0-2 = “no”  3-4 = “yes” | **Any purging (age 24)**  Any self-induced vomiting  OR  Any laxative use  OR  Any other medication use |
| During the **past year**, how often did you take laxatives to lose weight or avoid gaining weight? | | 1. Never 2. Less than once a month 3. 1-3 times a month 4. Once a week 5. More than once a week | | Any laxative use  0 = “no”  1-4 = “yes”  DSM laxative use  0-2 = “no”  3-4 = “yes” |  |
| During the **past year**, how often did you take other tablets/pills/any other medications or substances to lose weight or avoid gaining weight? | | 1. Never 2. Less than once a month 3. 1-3 times a month 4. Once a week 5. More than once a week | | Any other medication use  0 = “no”  1-4 = “yes”  DSM other medication use  0-2 = “no”  3-4 = “yes” |  |

| **Questions** | **Answers** | | **Coding** | | **Final variable** |
| --- | --- | --- | --- | --- | --- |
| **Excessive exercise (age 24)** | | | | | |
| During the **past year**, how often did you exercise to **lose weight** or **avoid gaining weight**? | | 1. Never 2. Less than once a month 3. 1-3 times a month 4. 1-4 times a week 5. 5 or more times a week | | Any exercise to lose weight  0 = “no”  1-4 = “yes”  DSM exercise to lose weight  0-2 = “no”  3-4 = “yes” | **Any excessive exercise (age 24)**  Any exercise to lose weight  AND  Exercise interfered with life |
| Was it difficult for you to do your work or daily chores/routine because of the amount of time that you were exercising to lose weight or avoid gaining weight? | | 1. No 2. Yes, sometimes 3. Yes, frequently | | Exercise interfered with life  0-1 = “no”  2 = “yes” |  |
| **Binge eating (age 24)** | | | | | |
| Sometimes people will go on an ‘eating binge’, where they eat an amount of food that most people, like their friends or family, would consider to be very large in a short period of time. During the **past year**, how often did you go on an eating binge? | | 1. Never 2. Less than once a month 3. 1-3 times a month 4. Once a week 5. More than once a week | | Any bingeing  0 = “no”  1-4 = “yes”  DSM bingeing  0-2 = “no”  3-4 = “yes” | **Any binge eating (age 24)**  Any bingeing  AND  Loss of control |
| Do you ever feel like your eating is out of control, like you couldn’t stop eating even if you wanted to? | | 1. No 2. Yes, sometimes 3. Yes, usually | | Loss of control  0 = “no”  1-2 = “yes” |  |
| **Any disordered eating (age 24)** | | | | | |
|  | |  | |  | **Any disordered eating (age 24)**  Any fasting OR  Any purging OR  Any binge-eating OR  Any excessive exercise |
| **DSM-5 disordered eating (age 24)** | | | | | |
|  | |  | | **DSM purging**  DSM self-induced vomiting OR  DSM laxative use OR  DSM Other medication use  **DSM binge-eating**  DSM bingeing AND  Loss of control  **DSM excessive exercise**  DSM exercise to lose weight AND  Exercise interfered with life | **DSM-5 disordered eating (age 24)**  DSM fasting  OR  DSM purging  OR  DSM binge-eating  OR  DSM excessive exercise |
| **Self-harm (age 24)** | | | | | |
| Have you **ever** hurt yourself on purpose in any way (e.g. by taking an overdose of pills or by cutting yourself)? | | 1. No 2. Yes | | Self-harm ever  0 = “no”  1 = “yes” | **Self-harm (age 24)**  Self-harm past year  (= 0 if self-harm ever = 0) |
| **If yes,** how many times have you done this in the last year? | | 1. None 2. Once 3. 2-5 times 4. 6-10 times 5. More than 10 times | | Self-harm past year  0 = “no”  1-4 = “yes” |  |

**Table S2. Prevalence of disordered eating and self-harm at 16 and 24 years across different analytical samples**

|  | Female | | | | | Male | | | | |
| --- | --- | --- | --- | --- | --- | --- | --- | --- | --- | --- |
|  | Sample 1 (n=1798) | | Sample 2 (n16=2699, n24=2483) | | Sample 3 (MI sample, n=3384) | Sample 1 (n=915) | | Sample 2 (n16=1882, n24=1359) | | Sample 3 (MI sample,  n=2326) |
|  | N | % (se) | N | % (se) | % (se) | N | % (se) | N | % (se) | % (se) |
| *Age 16* |  |  |  |  |  |  |  |  |  |  |
| Any fasting | 343 | 19.1% (0.93) | 544 | 20.2% (0.77) | 20.7% (0.77) | 23 | 2.5% (0.52) | 57 | 3.0% (0.40) | 3.4% (0.45) |
| Any purging | 157 | 8.7% (0.67) | 250 | 9.3% (0.56) | 9.5% (0.56) | ** | ** | 22 | 1.2% (0.25) | 1.9% (0.38) |
| Any binge-eating | 280 | 15.6% (0.86) | 426 | 15.8% (0.70) | 16.2% (0.70) | 39 | 4.3% (0.67) | 73 | 3.9% (0.45) | 4.3% (0.48) |
| Any excessive exercise | 33 | 1.8% (0.32) | 53 | 2.0% (0.27) | 2.1% (0.28) | ** | ** | 7 | 0.4% (0.14) | 0.8% (0.27) |
| Any disordered eating behaviour | 557 | 31.0% (1.10) | 866 | 32.1% (0.90) | 32.7% (0.89) | 62 | 6.8% (0.83) | 134 | 7.1% (0.59) | 7.6% (0.63) |
| Any disordered eating behaviour at DSM 5 level frequency | 175 | 9.7% (0.70) | 287 | 10.6% (0.59) | 11.1% (0.61) | 15 | 1.6% (0.42) | 41 | 2.2% (0.34) | 2.8% (0.41) |
| Any self-harm | 256 | 14.2% (0.82) | 404 | 15.0% (0.69) | 15.3% (0.68) | 49 | 5.4% (0.74) | 101 | 5.4% (0.52) | 5.4% (0.53) |
|  |  |  |  |  |  |  |  |  |  |  |
| *Age 24* |  |  |  |  |  |  |  |  |  |  |
| Any fasting | 216 | 12.0% (0.77) | 326 | 13.1% (0.68) | 13.6% (0.67) | 44 | 4.8% (0.71) | 71 | 5.2% (0.60) | 6.2% (0.70) |
| Any purging | 220 | 12.2% (0.77) | 307 | 12.4% (0.66) | 12.7% (0.67) | 16 | 1.8% (0.43) | 30 | 2.2% (0.40) | 3.2% (0.55) |
| Any binge-eating | 426 | 23.7% (1.00) | 610 | 24.6% (0.86) | 24.8% (0.86) | 118 | 12.9% (1.11) | 181 | 13.3% (0.92) | 13.7% (0.97) |
| Any excessive exercise | 18 | 1.0% (0.23) | 30 | 1.2% (0.22) | 1.6% (0.28) | ** | ** | 12 | 0.9% (0.25) | 2.0% (0.47) |
| Any disordered eating behaviour | 615 | 34.2% (1.12) | 904 | 36.4% (0.97) | 36.9% (0.96) | 158 | 17.3% (1.25) | 252 | 18.5% (1.05) | 19.2% (1.02) |
| Any disordered eating behaviour at DSM 5 level frequency | 178 | 9.9% (0.70) | 274 | 11.0% (0.63) | 11.3% (0.63) | 34 | 3.7% (0.63) | 71 | 5.2% (0.60) | 5.9% (0.66) |
| Any self-harm | 174 | 9.7% (0.70) | 238 | 9.6% (0.59) | 9.8% (0.61) | 43 | 4.7% (0.70) | 59 | 4.3% (0.55) | 5.1% (0.68) |

**Data censored to maintain participant anonymity (cell count ≤5)

**Table S3.1. Differences between respondents and non-respondents predicted by imputation model (age 16)**

|  | Female | | | Male | | |
| --- | --- | --- | --- | --- | --- | --- |
|  | Observed data (n=2699) | Imputed  data (n=685)  % (se) | Risk  Ratio  [95% CI] | Observed data (n=1882) | Imputed data (n=444) | Risk  Ratio  [95% CI] |
|  | % (se) |  |  | % (se) | % (se) |  |
| Any fasting | 20.2% (0.77) | 22.6% (2.28) | 1.12 [0.91, 1.39] | 3.0% (0.40) | 4.9% (0.40) | 1.62 [0.80, 1.39] |
| Any purging | 9.3% (0.56) | 10.3% (1.71) | 1.11 [0.79, 1.57] | 1.2% (0.25) | 4.7% (1.68) | 4.06 [1.81, 9.12] |
| Any binge-eating | 15.8% (0.70) | 17.8% (2.13) | 1.13 [0.88, 1.44] | 3.9% (0.45) | 5.7% (1.65) | 1.48 [0.81, 2.72] |
| Any excessive exercise | 2.0% (0.27) | 2.7% (0.91) | 1.38 [0.68, 2.81] | 0.4% (0.14) | 2.6% (1.29) | 6.90 [2.00, 23.8] |
| Any disordered eating behaviour | 32.1% (0.90) | 35.2% (2.62) | 1.10 [0.94, 1.28] | 7.1% (0.59) | 9.5% (2.13) | 1.33 [0.83, 2.13] |
| Any disordered eating behaviour at DSM 5 level frequency | 10.6% (0.59) | 12.9% (1.88) | 1.22 [0.90, 1.65] | 2.2% (0.34) | 5.1% (1.64) | 2.34 [1.16, 4.71] |
| Any self-harm | 15.0% (0.69) | 16.6% (2.00) | 1.11 [0.86, 1.43] | 5.4% (0.52) | 5.7% (1.65) | 1.07 [0.59, 1.94] |

Risk Ratio quantifies the extent to which non-respondents are at greater risk of each variable compared to respondents

**Table S3.2. Differences between respondents and non-respondents predicted by imputation model (age 24)**

|  |  | Female |  |  | Male |  |
| --- | --- | --- | --- | --- | --- | --- |
|  | Observed data (n=2483)  % (se) | Imputed  data (n=901)  % (se) | Risk  Ratio  [95% CI] | Observed data (n=1359)  % (se) | Imputed data (n=967)  % (se) | Risk  Ratio  [95% CI] |
|  |  |  |  |  |  |  |
| Any fasting | 13.1% (0.68) | 14.8% (1.66) | 1.13 [0.89, 1.44] | 5.2% (0.60) | 7.6% (1.45) | 1.46 [0.95, 2.26] |
| Any purging | 12.4% (0.66) | 13.4% (1.74) | 1.09 [0.82, 1.43] | 2.2% (0.40) | 4.6% (1.22) | 2.08 [1.11, 3.93] |
| Any binge-eating | 24.6% (0.86) | 25.5% (2.19) | 1.04 [0.86, 1.24] | 13.3% (0.92) | 14.2% (1.94) | 1.06 [0.79, 1.44] |
| Any excessive exercise | 1.2% (0.22) | 2.5% (0.91) | 2.06 [0.93, 4.60] | 0.9% (0.25) | 3.6% (1.06) | 4.09 [1.83, 9.16] |
| Any disordered eating behaviour | 36.4% (0.97) | 38.2% (2.46) | 1.05 [0.91, 1.20] | 18.5% (1.05) | 20.1% (1.95) | 1.08 [0.87, 1.35] |
| Any disordered eating behaviour at DSM 5 level frequency | 11.0% (0.63) | 12.0% (1.63) | 1.09 [0.81, 1.45] | 5.2% (0.60) | 6.8% (1.34) | 1.29 [0.82, 2.03] |
| Any self-harm | 9.6% (0.59) | 10.5% (1.63) | 1.09 [0.78, 1.52] | 4.3% (0.55) | 6.2% (1.44) | 1.42 [0.84, 2.39] |

Risk Ratio quantifies the extent to which non-respondents are at greater risk of each variable compared to respondents

**Table S4.1. Rates of self-harm across strata of disordered eating (age 16)**

|  | Female | | | Male | | |
| --- | --- | --- | --- | --- | --- | --- |
|  | Sample 1 (n=1798) | Sample 2 (n=2699) | Sample 3 (MI sample, n=3384) | Sample 1 (n=915) | Sample 2 (n=1882) | Sample 3 (MI sample,  n=2326) |
|  | % (se) /  RR [95% CI] | % (se) /  RR [95% CI] | % (se) /  RR [95% CI] | % (se) /  RR [95% CI] | % (se) /  RR [95% CI] | % (se) /  RR [95% CI] |
| Any fasting |  |  |  |  |  |  |
| No | 9.6% (0.77) | 10.1% (0.65) | 10.2% (0.66) | 4.6% (0.70) | 4.5% (0.48) | 4.5% (0.49) |
| Yes | 33.8% (2.55) | 34.4% (2.04) | 35.0% (2.00) | 34.8% (9.93) | 33.3% (6.24) | 32.1% (6.21) |
|  | 3.51 [2.83, 4.36] | 3.41 [2.88, 4.05] | 3.44 [2.90, 4.08] | 7.57 [4.01, 14.3] | 7.42 [4.86, 11.3] | 7.13 [4.58, 11.1] |
| Any purging |  |  |  |  |  |  |
| No | 11.6% (0.79) | 12.0% (0.66) | 12.2% (0.66) | 5.3% (0.74) | 5.0% (0.51) | 5.1% (0.51) |
| Yes | 41.4% (3.93) | 44.4% (3.14) | 45.4% (3.09) | ** | 36.4% (10.3) | 34.4% (9.49) |
|  | 3.56 [2.83, 4.47] | 3.71 [3.11, 4.42] | 3.72 [3.14, 4.41] | ** | 7.27 [4.04, 13.1] | 6.81 [3.81, 12.2] |
| Any binge-eating | |  |  |  |  |  |
| No | 12.1% (0.84) | 12.7% (0.70) | 12.9% (0.69) | 4.7% (0.71) | 4.8% (0.50) | 4.9% (0.52) |
| Yes | 25.7% (2.61) | 27.2% (2.16) | 28.2% (2.14) | 20.5% (6.47) | 19.2% (4.61) | 19.4% (4.73) |
|  | 2.12 [1.67, 2.70] | 2.15 [1.78, 2.60] | 2.19 [1.82, 2.63] | 4.38 [2.21, 8.71] | 3.99 [2.39, 6.66] | 3.95 [2.33, 6.69] |
| Any excessive exercise | |  |  |  |  |  |
| No | 14.1% (0.83) | 14.6% (0.69) | 14.9% [0.68] | 5.3% (0.74) | 5.3% (0.52) | 5.4% (0.51) |
| Yes | 24.2% (7.46) | 34.0% (6.51) | 34.9% (6.54) | ** | ** | 28.7% (16.8) |
|  | 1.73 [0.93, 3.19] | 2.33 [1.58, 3.43] | 2.33 [1.60, 3.41] | ** | ** | 5.32 [1.63, 17.4] |
| Any disordered eating behaviour | |  |  |  |  |  |
| No | 7.6% (0.75) | 8.2% (0.64) | 8.3% (0.65) | 3.9% (0.66) | 3.9% (0.47) | 4.0% (0.47) |
| Yes | 29.1% (1.92) | 29.3% (1.55) | 29.9% (1.52) | 25.8% (5.56) | 23.9% (3.68) | 23.7% (3.64) |
|  | 3.84 [3.04, 4.85] | 3.58 [3.00, 4.31] | 3.62 [3.00, 4.36] | 6.67 [3.89, 11.4] | 6.05 [4.13, 8.85] | 5.93 [4.05, 8.70] |
| Any disordered eating behaviour at DSM 5 level frequency | | | |  |  |  |
| No | 12.1% (0.81) | 12.4% (0.67) | 12.7% (0.66) | 5.1% (0.73) | 4.9% (0.51) | 5.0% (0.52) |
| Yes | 33.7% (3.57) | 36.2% (2.84) | 37.0% (2.83) | ** | 24.4% (6.71) | 24.2% (6.47) |
|  | 2.78 [2.17, 3.55] | 2.91 [2.42, 3.51] | 2.92 [2.44, 3.50] | ** | 4.93 [2.78, 8.77] | 4.85 [2.78, 8.48] |

**Data censored to maintain participant anonymity (cell count ≤5)

**Table S4.2. Rates of self-harm across strata of disordered eating (age 24)**

|  | Female | | | Male | | |
| --- | --- | --- | --- | --- | --- | --- |
|  | Sample 1 (n=1798) | Sample 2 (n=2483) | Sample 3 (MI sample, n=3384) | Sample 1 (n=915) | Sample 2 (n=1359) | Sample 3 (MI sample,  n=2326) |
|  | % (se) /  RR [95% CI] | % (se) /  RR [95% CI] | % (se) /  RR [95% CI] | % (se) /  RR [95% CI] | % (se) /  RR [95% CI] | % (se) /  RR [95% CI] |
| Any fasting |  |  |  |  |  |  |
| No | 7.9% (0.68) | 7.6% (0.57) | 7.8% (0.58) | 3.7% (0.64) | 3.6% (0.52) | 4.3% (0.64) |
| Yes | 22.7% (2.85) | 23.1% (2.33) | 23.0% (2.34) | 25.0% (6.53) | 18.3% (4.59) | 17.9% (4.48) |
|  | 2.87 [2.12, 3.87] | 3.04 [2.38, 3.90] | 2.97 [2.32, 3.78] | 6.80 [3.68, 12.6] | 5.13 [2.91, 9.04] | 4.22 [2.39, 7.46] |
| Any purging |  |  |  |  |  |  |
| No | 8.4% (0.70) | 8.0% (0.58) | 8.2% (0.59) | 4.6% (0.70) | 4.2% (0.55) | 5.0% (0.64) |
| Yes | 19.1% (2.65) | 20.5% (2.30) | 20.7% (2.31) | ** | ** | 9.7% (5.06) |
|  | 2.28 [1.66, 3.13] | 2.55 [1.96, 3.32] | 2.52 [1.94, 3.29] | ** | ** | 1.96 [0.68, 5.63] |
| Any binge-eating | |  |  |  |  |  |
| No | 8.1% (0.74) | 7.8% (0.62) | 8.0% (0.62) | 4.0% (0.70) | 3.7% (0.55) | 4.4% (0.68) |
| Yes | 14.8% (1.72) | 14.9% (1.44) | 15.1% (1.44) | 9.3% (0.27) | 8.8% (2.11) | 9.3% (2.23) |
|  | 1.82 [1.37, 2.44] | 1.90 [1.49, 2.43] | 1.89 [1.49, 2.40] | 2.32 [1.20, 4.48] | 2.42 [1.39, 4.21] | 2.12 [1.23, 3.65] |
| Any excessive exercise | |  |  |  |  |  |
| No | 9.5% (0.69) | 9.4% (0.59) | 9.6% (0.59) | 4.7% (0.70) | 4.3% (0.55) | 5.1% (0.68) |
| Yes | ** | 23.3% (7.72) | 23.2% (7.37) | ** | ** | 10.0% (9.82) |
|  | ** | 2.48 [1.28, 4.79] | 2.42 [1.28, 4.57] | ** | ** | 1.97 [0.28, 13.9] |
| Any disordered eating behaviour | |  |  |  |  |  |
| No | 6.5% (0.72) | 6.0% (0.60) | 6.0% (0.62) | 3.2% (0.64) | 3.0% (0.51) | 3.6% (0.65) |
| Yes | 15.8% (1.47) | 15.9% (1.22) | 16.1% (1.19) | 12.0% (2.59) | 10.3% (1.91) | 11.1% (2.05) |
|  | 2.42 [1.83, 3.22] | 2.68 [2.09, 3.42] | 2.67 [2.09, 3.42] | 3.79 [2.13, 6.75] | 3.46 [2.11, 5.68] | 3.06 [1.83, 5.11] |
| Any disordered eating behaviour at DSM 5 level frequency | | | |  |  |  |
| No | 8.6% (0.70) | 8.2% (0.59) | 8.4% (0.59) | 4.3% (0.68) | 3.9% (0.54) | 4.5% (0.62) |
| Yes | 19.1% (2.95) | 20.4% (2.44) | 20.7% (2.46) | ** | 12.7% (0.39) | 13.8% (4.01) |
|  | 2.21 [1.57, 3.11] | 2.48 [1.89, 3.26] | 2.47 [1.89, 3.24] | ** | 3.27 [1.67, 6.37] | 3.10 [1.66, 5.79] |

**Data censored to maintain participant anonymity (cell count ≤5)

**Table S5. Rates of disordered eating across strata of self-harm**

|  |  |  | Females | | | Males | | |
| --- | --- | --- | --- | --- | --- | --- | --- | --- |
|  |  |  | Sample 1 (n=1798) | Sample 2 (n16=2699,  n24=2483) | Sample 3 (MI sample, n=3384) | Sample 1 (n=915) | Sample 2 (n16=1882,  n24=1359) | Sample 3 (MI sample,  n=2326) |
| DE measure | Age | SH status | % (se) /  RR [95% CI] | % (se) /  RR [95% CI] | % (se) /  RR [95% CI] | % (se) /  RR [95% CI] | % (se) /  RR [95% CI] | % (se) /  RR [95% CI] |
| Any fasting | Age 16 | No | 14.7% (0.90) | 15.6% (0.76) | 15.9% (0.75) | 1.7% (0.44) | 2.1% (0.34) | 2.4% (0.39) |
|  |  | Yes | 45.3% (3.11) | 46.3% (2.48) | 47.2% (2.51) | 16.3% (5.28) | 18.8% (3.89) | 20.1% (4.23) |
|  |  |  | 3.08 [2.57, 3.69] | 2.98 [2.58, 3.43] | 2.98 [2.59, 3.42] | 9.43 [4.20, 21.2] | 8.82 [5.28, 14.7] | 8.23 [4.90, 13.8] |
|  | Age 24 | No | 10.3% (0.75) | 11.2% (0.67) | 11.6% (0.67) | 3.8% (0.64) | 4.5% (0.57) | 5.4% (0.68) |
|  |  | Yes | 28.2% (3.41) | 31.5% (3.01) | 31.8% (2.94) | 25.6% (6.65) | 22.0% (5.40) | 21.9% (5.23) |
|  |  |  | 2.74 [2.06, 3.61] | 2.82 [2.26, 3.51] | 2.74 [2.21, 3.41] | 6.76 [3.67, 12.4] | 4.94 [2.87, 8.49] | 4.06 [2.36, 6.99] |
| Any purging | Age 16 | No | 6.0% (0.60) | 6.1% (0.50) | 6.1% (0.50) | ** | 0.8% (0.21) | 1.3% (0.32) |
|  |  | Yes | 25.4% (2.72) | 27.5% (2.22) | 28.0% (2.21) | ** | 7.9% (2.69) | 11.5% 93.68) |
|  |  |  | 4.26 [3.19, 5.68] | 4.54 [3.62, 5.69] | 4.59 [3.67, 5.73] | ** | 10.1 [4.33, 23.5] | 8.93 [4.06, 19.6] |
|  | Age 24 | No | 11.0% (0.78) | 10.9% (0.66) | 11.1% (0.68) | 1.6% (0.43) | 2.1% (0.40) | 3.0% (0.55) |
|  |  | Yes | 24.1% (3.24) | 26.5% (2.86) | 26.8% (2.85) | ** | ** | 6.1% (3.23) |
|  |  |  | 2.20 [1.64, 2.97] | 2.44 [1.91, 3.10] | 2.41 [1.88, 3.07] | ** | ** | 2.01 [0.67, 6.00] |
| Any binge-eating | Age 16 | No | 13.5% (0.87) | 13.5% (0.71) | 13.7% (0.71) | 3.6% (0.63) | 3.3% (0.42) | 3.6% (0.47) |
|  |  | Yes | 28.1% (2.81) | 28.7% (2.25) | 29.7% (2.23) | 16.3% (5.28) | 13.9% (3.44) | 14.9% (3.64) |
|  |  |  | 2.09 [1.65, 2.63] | 2.13 [1.77, 2.58] | 2.16 [1.81, 2.58] | 4.56 [2.22, 9.39] | 4.18 [2.42, 7.23] | 4.12 [2.36, 7.19] |
|  | Age 24 | No | 22.4% (1.03) | 23.1% (0.89) | 23.3% (0.89) | 12.3% (1.11) | 12.7% (0.92) | 13.0% (0.96) |
|  |  | Yes | 36.2% (3.64) | 38.2% (3.15) | 38.4% (3.08) | 25.6% (6.65) | 27.1% (5.78) | 25.1% (5.49) |
|  |  |  | 1.62 [1.30, 2.01] | 1.65 [1.38, 1.98] | 1.65 [1.38, 1.96] | 2.08 [1.22, 3.58] | 2.14 [1.37, 3.32] | 1.92 [1.23, 2.99] |

**Data censored to maintain participant anonymity (cell count ≤5)

**Table S5. Rates of disordered eating across strata of self-harm (cont.)**

|  |  |  | Females | | | Males | | |
| --- | --- | --- | --- | --- | --- | --- | --- | --- |
|  |  |  | Sample 1 (n=1798) | Sample 2 (n16=2699,  n24=2483) | Sample 3 (MI sample, n=3384) | Sample 1 (n=915) | Sample 2 (n16=1882,  n24=1359) | Sample 3 (MI sample,  n=2326) |
| DE measure | Age | SH status | % (se) /  RR [95% CI] | % (se) /  RR [95% CI] | % (se) /  RR [95% CI] | % (se) /  RR [95% CI] | % (se) /  RR [95% CI] | % (se) /  RR [95% CI] |
| Any excessive exercise | Age 16 | No | 1.6% (0.32) | 1.5% (0.26) | 1.6% (0.26) | ** | ** | 0.6% (0.24) |
|  |  | Yes | 3.1% (0.11) | 4.5% (1.03) | 4.8% (1.13) | ** | ** | 4.1% (2.83) |
|  |  |  | 1.93 [0.88, 4.23] | 2.92 [1.67, 5.11] | 2.96 [1.70, 5.15] | ** | ** | 7.17 [1.42, 36.2] |
|  | Age 24 | No | 0.8% (0.22) | 1.0% (0.21) | 1.3% (0.27) | ** | 0.8% (0.25) | 1.8% (0.46) |
|  |  | Yes | ** | 2.9% (1.10) | 3.7% (1.32) | ** | ** | 3.9% (3.93) |
|  |  |  | ** | 2.87 [1.25, 6.62] | 2.80 [1.26, 6.21] | ** | ** | 2.11 [0.25, 17.4] |
| Any disordered eating behaviour | Age 16 | No | 25.6% (1.11) | 26.7% (0.92) | 27.1% (0.93) | 5.3% (0.76) | 5.7% (0.55) | 6.1% (0.59) |
|  |  | Yes | 63.3% (3.01) | 62.9% (2.40) | 63.7% (2.37) | 32.7% (6.70) | 31.7% (4.63) | 32.7% (4.68) |
|  |  |  | 2.47 [2.18, 2.80] | 2.36 [2.13, 2.61] | 2.35 [2.12, 2.60] | 6.15 [3.76, 10.0] | 5.53 [3.93, 7.79] | 5.35 [3.81, 7.52] |
|  | Age 24 | No | 31.9% (1.16) | 33.9% (1.00) | 34.3% (0.99) | 15.9% (1.24) | 17.4% (1.05) | 18.0% (1.04) |
|  |  | Yes | 55.8% (3.77) | 60.5% (3.17) | 60.9% (3.18) | 44.2% (7.57) | 44.1% (6.46) | 41.9% (6.42) |
|  |  |  | 1.75 [1.50, 2.03] | 1.77 [1.59, 2.01] | 1.78 [1.58, 2.00] | 2.77 [1.92, 4.01] | 2.53 [1.86, 3.46] | 2.33 [1.69, 3.26] |
| Any disordered eating behaviour at DSM 5 level frequency | Age 16 | No | 7.5% (0.67) | 8.0% (0.57) | 8.3% (0.58) | 1.4% (0.40) | 1.7% (0.31) | 2.2% (0.38) |
|  |  | Yes | 23.0% (2.63) | 25.7% (2.18) | 26.7% (2.18) | ** | 9.9% (2.97) | 12.1% (3.43) |
|  |  |  | 3.06 [2.31, 4.07] | 3.23 [2.60, 4.01] | 3.23 [2.62, 3.99] | ** | 5.69 [2.87, 11.3] | 5.49 [2.86, 10.5] |
|  | Age 24 | No | 8.9% (0.71) | 9.7% (0.62) | 9.9% (0.63) | 3.3% (0.61) | 4.8% (0.59) | 5.3% (0.65) |
|  |  | Yes | 19.5% (3.01) | 23.5% (2.75) | 24.0% (2.75) | ** | 15.3% (4.68) | 16.2% (4.58) |
|  |  |  | 2.20 [1.57, 3.09] | 2.42 [1.87, 3.15] | 2.41 [1.86, 3.12] | ** | 3.20 [1.67, 6.12] | 3.05 [1.66, 5.59] |

**Data censored to maintain participant anonymity (cell count ≤5)

**Table S6.1 Complete case (Sample 1) descriptive analysis for any disordered eating in those with no self-harm, a single self-harm instance and repeated self-harm instances at age 16**

|  |  | Females (n=1797) | | | Males (n=915) | | |
| --- | --- | --- | --- | --- | --- | --- | --- |
|  |  | No self-harm  N (%) | 1 instance self-harm  N (%) | 2+ instances self-harm  N (%) | No self-harm  N (%) | 1 instance self-harm  N (%) | 2+ instances self-harm  N (%) |
| Any disordered eating | No | 1073 (76.15%) | 83 (56.46%) | 84 (34.85%) | 798 (95.11%) | 25 (71.43%) | 30 (73.17%) |
|  | Yes | 336 (23.85%) | 64 (43.54%) | 157 (65.15%) | 41 (4.89%) | 10 (28.57%) | 11 (26.83%) |

*Note: 1 female did not disclose frequency of self-harm in the last year*

**Table S6.2 Complete case (Sample 1) descriptive analysis for any disordered eating in those with no self-harm, a single self-harm instance and repeated self-harm instances at age 24**

|  |  | Females (n=1798) | | | Males (n=915) | | |
| --- | --- | --- | --- | --- | --- | --- | --- |
|  |  | No self-harm  N (%) | 1 instance self-harm  N (%) | 2+ instances self-harm  N (%) | No self-harm  N (%) | 1 instance self-harm  N (%) | 2+ instances self-harm  N (%) |
| Any disordered eating | No | 1106 (68.10%) | 29 (51.79%) | 48 (40.68%) | 733 (84.06%) | 7 (53.85%) | 17 (56.67%) |
|  | Yes | 518 (31.90%) | 27 (48.21%) | 70 (59.32%) | 139 (15.94%) | 6 (46.15%) | 13 (43.33%) |
